# Predictors of instanteous relief from spinal manipulation for non-specific low back pain: a delphi study

**DOI:** 10.1186/s12998-020-00324-7

**Published:** 2020-07-02

**Authors:** Stanley Innes, Amber Beynon, Christopher Hodgetts, Rachel Manassah, Denyse Lim, Bruce F. Walker

**Affiliations:** 1 Psychology, Exercise Science, Counselling and Chiropractic, Murdoch University, Murdoch, Australia; 2 5th Year student, Psychology, Exercise Science, Counselling and Chiropractic, Murdoch University, Murdoch, Australia

**Keywords:** Spinal manipulation, Delphi, Instantaneous response

## Abstract

**Background:**

There is some evidence and anecdotal reports that high-velocity low-amplitude (HVLA) spinal manipulation therapy (SMT) for non-specific low back pain (NSLBP) may immediately reduce pain in some patients. The mechanism for such a change remains unclear and the evidence is conflicting. The aim of this study was to seek consensus among a sample of expert manual therapists as to the possible clinical predictors that could help identify patients who are most likely to receive instant relief from NSLBP with SMT intervention.

**Methods:**

Thirty-seven expert chiropractors and manipulative physiotherapists from around the world were invited to participate in a three round online Delphi questionnaire during the second half of 2018. Participants were provided with a list of 55 potential signs and symptoms as well as offering them the option of suggesting other factors in the first round. The variables were rated using a 4-point Likert likelihood scale and a threshold of 75% agreement was required for any item to progress to the next round.

**Results:**

Of these 37 experts, 19 agreed to participate. Agreement as to the proportion of patients who receive instantaneous relief was minimal (range 10–80%). A total of 62 items were ranked over the 3 rounds, with 18 of these retained following the third round. The highest rated of the 18 was ‘A history including a good response to previous spinal manipulation’.

**Discussion/conclusion:**

Five categories; patient factors, practitioner factors, signs and symptoms of NSLBP presentation, an instrument of measurement (FABQ), and the presence of cavitation following SMT best describe the overall characteristics of the factors. The 18 factors identified in this study can potentially be used to create an instrument of measurement for further study to predict those patients with NSLBP who will receive instantaneous relief post-SMT.

## Background

Physiotherapists, osteopaths and chiropractors are among health professions with a special interest in the diagnosis, management and prevention of musculoskeletal disorders, especially back pain [1, 2]. Patients suffering from musculoskeletal conditions are often treated using manual therapy by these practitioners. A common intervention used by these practitioners are spinal manipulation techniques (SMT) [2–4]. The current literature and anecdotal evidence in clinical practice suggests that SMT is utilized with the aim of improving joint ranges of motion, releasing muscular tension in order to improve joint function and decrease or relieve musculoskeletal pain [5, 6].

Some studies suggest that the mechanical force induced by an effective high-velocity low-amplitude (HVLA) technique to a specific spinal segment can induce immediate pain relief [7, 8], others disagree [9]. The literature posits a number of theories to explain this possible outcome, including but not limited to, neurophysiological and biomechanical effects [10], enhanced facet joint motion, intra-articular or myofascial adhesions, and soft tissue inclusions entrapped between facet joints [8, 9, 11–13]. Some suggest that this may not be exclusive to SMT [14, 15].

There has been a call to action by prominent researchers for a change in the way low back pain (LBP), one of the most commonly encountered musculoskeletal conditions [16], is managed [17], in an effort to reduce the huge financial impact placed on economies [18]. One recommendation was for the reduction of care that yields marginal benefits at a disproportionately high cost (low value care) [17]. In support of this concern is recent research that shows that chiropractic students are not good at predicting when SMT will not make a difference to patient outcomes [19, 20]. Given that any intervention should be targeted to those who are likely to gain the most benefit, it logically follows that knowing the clinical predictors is important when selecting patients for HVLA SMT [21]. It is hypothesized and anecdotally reported, that there is a subgroup of the population who respond to spinal manipulation with instantaneous relief, and that these patients can be identified prior to treatment [7]. It is known that the most favourable prognostic group undergoing chiropractic care (common users of HVLA SMT) at the fourth consultation are those who respond strongly after the first consultation [22, 23]. However, understanding and identifying who is included in this population is poorly reported in the literature and not well researched [7]. Chiropractors have been shown to be poor predictors of those most likely to respond positively to care [24]. Thus the ability to identify a group of people who respond strongly and immediately to SMT could contribute to the reduction of low value care and improve the quality of patient care.

The aim of this study was to seek consensus among a sample of expert manual therapists as to the possible clinical predictors that could help identify patients who are most likely to receive instant relief from NSLBP with SMT intervention.

## Methods

### Rationale for Delphi technique

To address our research question a Delphi technique was chosen because the technique is considered to have a qualitative dimension that is appropriate when quantitative methods are unlikely to yield results that can be relatively easily gathered or be readily implemented into practice [25]. The Delphi technique employed for this study used a literature review to create the initial item list and a focus group to review and pilot test the survey.

### Development of the questionnaire

The questionnaire was developed in two stages: 1) item generation 2) item selection.

#### Stage 1. Item generation

The items were generated via a review and analysis of the literature looking for signs and symptoms possibly linked to immediate relief from NSLBP following SMT. The literature search strategy used the MeSH and non-MeSH keywords: spinal manipulation, instant relief, immediate relief/response, non-specific low back pain, predictors of instant relief, predictors of immediate relief, predictors, indicators, and prevalence. Databases used were Google Scholar, PubMed, and Cochrane Library.

A focus group comprised of three manual therapy academics (BFW, AB, CH) and seven final year chiropractic students reviewed the item list for interpretability and created some additional items based on biological and clinical plausibility. This resulted in a list of 55 items (Table 1).

**Table 1:** The initial 55 item list of patient characteristics to predict patients likely to obtain immediate relief from NSLBP following SMT

**1**. Duration of symptoms < 16 days	**2**. Pain associated with coughing or sneezing
**3.** Fear Avoidance Beliefs Questionnaire work scale score less than 19 out of a high of 42 (low fear category)	**4.** A history including a good response to previous spinal manipulation
**5**. At least one hip with > 35 degrees of internal rotation	**6**. Patient has an acute condition (< 14 days)
**7**. Hypomobility in the lumbar spine	**8**. Patient has a subacute condition (> 3 months)
**9**. No symptoms distal to the knee	**10**. Patient has a chronic condition (> 3 months)
**11**. Morning stiffness ≤30 mins	**12**. Age 10–30
**13**. Morning stiffness > 30 mins	**14**. Age 31–49
**15**. Pain improves with exercise, but not rest	**16**. Age 50+
**17**. Pain on waking, duration > 30 mins	**18**. Female
**19**. Pain on waking, duration ≤30 mins	**20**. Male
**21**. Pain wakes the patient at night	**22**. Patient BMI < 35
**23**. Experiences stiffness after rest (gel phenomenon)	**24**. Patient BMI ≥ 35
**25**. Pain present at all times	**26**. Professional opinion of health status – fair/poor
**27**. Pain intermittently during the day	**28**. Professional opinion of health status – good
**29**. Pain develops later in the day	**30**. Professional opinion of health status – excellent/ very good
**31**. Pain associated with standing for a while	**32**. Patient experiencing depression
**33**. Pain associated with lifting	**34**. Patient experiences anxiety
**35**. Pain associated with bending forward a little	**36**. Patient is stressed
**37**. Pain associated with bending forward as far as they can	**38**. Good patient – practitioner relationship
**39**. Pain associated with arching backwards	**40**. Patient has a comprehensive understanding of condition
**41**. Pain associated with doing or attempting to do a sit up	**42**. Previous episode of NSLBP in patient history
**43**. Pain associated with driving long distances	**44**. Pain affecting activities of daily living
**45**. Pain associated with getting out of chair	**46**. Patient responds well to anti-inflammatory medicine
**47**. Pain associated with repetitive bending	**48**. Decreased active range of motion
**49**. Pain associated with running	**50**. Decreased passive range of motion
**51**. No symptoms in the lower extremities	**52**. Pain severity ≤5/10
**53**. Pain severity rated > 5/10	**54**. Recurrent attack of pain
**55**. First episode of pain	

#### Stage 2. Item selection

**Fig. 1:**
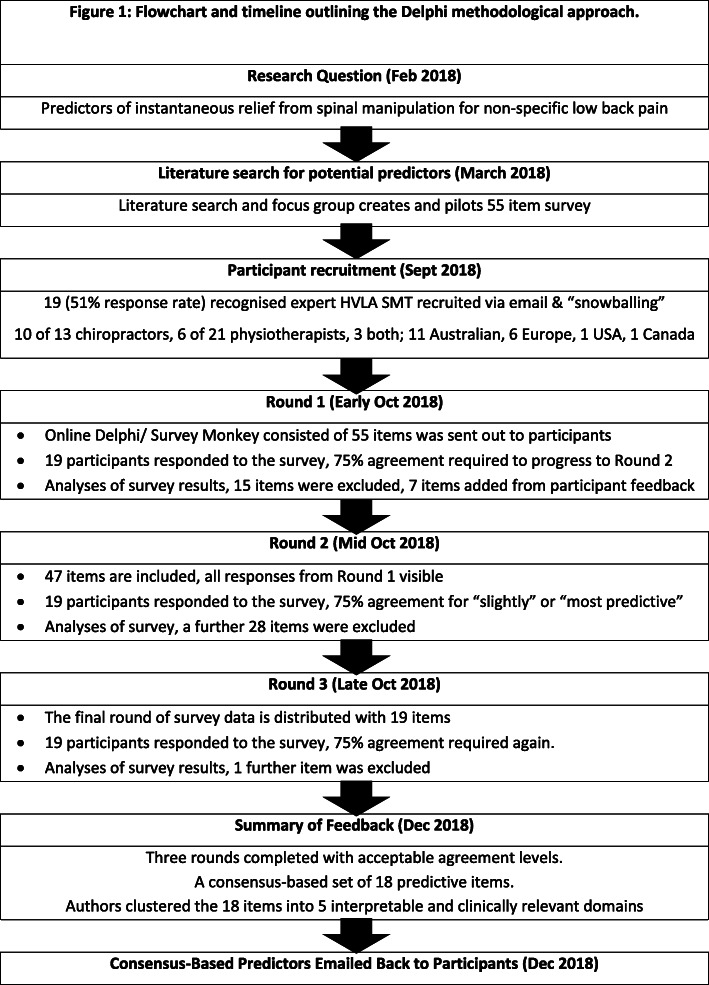
Flowchart and timeline outlining the Delphi methodological approach

A three round Delphi survey process [26, 27] was conducted by email correspondence to select and refine the collated items (see Fig. 1). The purpose of the survey was to see if any aspect that would lead to an item was overlooked, reach a consensus among the expert manual therapists regarding the signs and symptoms most likely to identify individuals who would respond immediately to HVLA SMT, and the likely percentage composition of daily practice this was thought to constitute.

### Participants: sample size, expert criteria and recruitment

Participants were selected on the basis of their relationship with the study phenomenon and following a criteria of suitability [28]. The criteria were 1) manual therapists registered as either chiropractors and/or manipulative physiotherapists with knowledge and interest in the subject as demonstrated by using HVLA SMT as a primary intervention for NSLBP; 2) had been using HVLA SMT for at least 5 years; 3) had experienced a patient response of immediate pain relief from HVLA SMT. Meeting these criteria was deemed to indicate that the practitioner participants had obtained a level of proficiency from clinical experience to have formed an opinion about NSLBP patients likely to benefit from SMT [26].

On the basis of past research, at least 7 to 10 experts from each of the chiropractic and physiotherapy disciplines needed to be recruited to obtain consensus [25, 26].

Recruitment began by emailing “experts” known to the authors for their clinical experience, after which a ‘snowball’ recruitment method was employed. The email contained a written invitation with an information letter explaining the methods and expectations. The manual therapists were also advised that they were participating in a peer group task endeavouring to work towards an end goal of consensus [27].

### Procedure for Delphi rounds and definition of consensus

An online survey (Survey Monkey [29]) was created to allow for easy access for all participants and efficient collection of data and could be completed in the consenting practitioners’ own time within 1 week. The methodology and timeline is outlined in Fig. 1.

#### Round 1

During the first round of the survey participants were blinded to their colleagues’ opinions and answered the survey anonymously. They were asked to rate the 55 items on a prediction scale that was structured as a modified 4-point Likert scale, which ranged from “Not predictive” to “Most predictive” (Additional file 1). A neutral response option was not provided as it would have given limited information to the research [27]. They were also invited to provide any additional signs or symptoms that may be applicable and had been overlooked. Responses were collated and reviewed by three of the authors (AB, DL, RM). Items where 75% of participants regarded the item to be ‘negatively predictive’ (not predictive or slightly un-predictive) were excluded from Round 2. Items that independently emerged from the open responses provided by practitioners were added to Round 2 and distributed again to the expert practitioners (Table 2).

**Table 2:** Shows the percentage of participants who responded “positively predictive” to the list of patient characteristics that may help predict instantaneous relief from NSLBP following SMT

No.	A list of patient characteristics that could help predict instantaneous relief from LBP following SMT	Percentage of participants who responded that the item is positively predictive (%)		
		Round 1	Round 2	Round 3
**1.**	Duration of symptoms < 16 days	94	94	88
**2.**	Morning stiffness ⩽ 30 mins	94	69	–
**3.**	A history including a good response to previous spinal manipulation	94	100	100
**4.**	Good patient-practitioner relationship	94	94	100
**5.**	No symptoms distal to the knee	88	88	82
**6.**	Patient has an acute condition (< 14 days)	88	94	82
**7.**	Professional opinion of health status - excellent/ very good	88	94	94
**8.**	Fear Avoidance Beliefs Questionnaire work scale score less than 19 out of a high of 42 (low fear category)	81	81	88
**9.**	Professional opinion of health status – good	81	88	82
**10**	Patient has a comprehensive understanding of condition	81	75	82
**11.**	Hypomobility in the lumbar spine	75	69	–
**12.**	Pain improves with exercise, but not rest	75	88	88
**13.**	Patient has a sub-acute condition (15 days to 3 months)	75	56	–
**14.**	Age 31–49	75	63	–
**15.**	Decreased active range of motion	75	81	82
**16.**	Pain intermittently during the day	69	63	–
**17.**	Previous episode of non-specific LBP in patient history	69	38	–
**18.**	Decreased passive range of motion	69	75	82
**19.**	No symptoms in the lower extremities	69	81	94
**20.**	Pain severity rated ⩽ 5/10	69	69	–
**21.**	Experiences stiffness after rest (gel phenomenon)	63	44	–
**22.**	Pain associated with getting out of a chair	63	44	–
**23.**	Pain affecting activities of daily living	63	44	–
**24.**	Pain associated with bending forward a little	56	38	–
**25.**	Pain associated with arching backwards	56	69	
**26.**	Age 10–30	56	44	–
**27.**	Recurrent attack of pain	50	38	–
**28.**	First episode of pain	50	25	–
**29.**	Pain associated with standing for a while	44	38	–
**30.**	Pain associated with lifting	44	13	–
**31.**	Pain associated with bending forward as far as they can	44	31	–
**32.**	Age 50+	44	19	–
**33.**	Pain on waking, duration ⩽ 30 mins	38	13	–
**34.**	At least one hip with > 35 degrees of internal rotation	31	13	–
**35.**	Pain on waking, duration > 30 mins	31	19	–
**36.**	Pain associated with repetitive bending	31	6	–
**37.**	Pain associated with running	31	19	–
**38.**	Patient BMI < 35	31	13	–
**39.**	Patient responds well to anti-inflammatory medicine	31	25	–
**40.**	Pain severity rated > 5/10	31	19	–
**41.**	Pain associated with doing or attempting to do a sit up	25	–	–
**42.**	Pain associated with driving long distances	25	–	–
**43.**	Female	25	–	–
**44.**	Male	25	–	–
**45.**	Pain present at all times	19	–	–
**46.**	Pain develops later in the day	19	–	–
**47.**	Pain associated with coughing or sneezing	19	–	–
**48.**	Morning stiffness > 30 mins	15	–	–
**49.**	Pain wakes the patient at night	13	–	–
**50.**	Patient has a chronic condition (> 3 months)	13	–	–
**51.**	Patient BMI ≥ 35	13	–	–
**52.**	Professional opinion of health status – fair/poor	7	–	–
**53.**	Patient experiencing depression	7	–	–
**54.**	Patient experiences anxiety	7	–	–
**55.**	Patient is stressed	7	–	–
**56.**	Pain onset related to a specific physical activity	n/a	56	–
**57.**	The production of a clicking sound (cavitation) at the moment of thrust	n/a	81	76
**58.**	Taking a comprehensive history	n/a	75	65
**59.**	Practitioner understanding of patient expectations and goals	n/a	88	94
**60.**	Close reproduction of symptoms on spinal springing and/ or end range loading	n/a	81	76
**61.**	Patient susceptible to placebo effect	n/a	88	88
**62.**	Patient has trust and confidence in the practitioner	n/a	81	100

**Key: n/a**: scores for these items are not available in Round 1 as they were added in Round 2 based on suggestions provided by participants in Round 1.

**-**: items that did not get scored as they had been removed from the survey.

#### Round 2

After the second round, the item list was once again revised by three members of the team (AB, DL, RM). This time items were retained if 75% of participants agreed they were positively predictive (most predictive or slightly predictive).

#### Round 3

For the Round 3 distribution, the predictor variables with 50% agreement or more as being ‘most predictive’ from Round 2 were ordered by desirability and placed at the top of the survey [25]. This revealed to the participant the popular and less popular options, giving them the opportunity to re-evaluate, re-think and either retain or change their original answer [26].

After three rounds, the four-point Likert scale of “not predictive”, “slightly un-predictive”, “slightly predictive” and “most predictive” was assigned a score of − 2, − 1, 1 and 2 respectively. This allowed for the final list of predictors that achieved a 75% level of agreement to be ranked based on their predictive scores by mean score and sum. A table was then generated allowing for notional weighting of the variables in order of their predictive score. This methodology ensured all possible options had been considered, estimated the consequential power of any particular option, and examined and estimated the acceptability of any particular option [28].

### Ethics

Sex, occupation and number of years of clinical experience were asked at the end of the survey. Participants remained anonymous and were not required to give any identifying information. Each participant received an online information letter and consent form (Additional file 1). The study was granted ethics approval by the Murdoch University Human Research Ethics Committee (2018/163).

## Results

### Participants

Thirty-seven experts (13 chiropractors, 21 physiotherapists, and 3 who were qualified as both a chiropractor and physiotherapist) were approached to participate. Of these 19 agreed to participate (51% response rate) comprising 10 chiropractors, 6 physiotherapists, and 3 who were qualified as both. The experts were from Australia (*n* = 11), Canada (*n* = 1), Denmark (*n* = 1), Germany (*n* = 1), Sweden (*n* = 1), United Kingdom (*n* = 3), and United States of America (*n* = 1).

### Round 1

The response rate for Round 1 was 18/19, Round 2 was 17/19 and Round 3 was 17/19. In Round 1 of the 18 participants who responded two were excluded as they responded positively to the exclusion question of not having observed an immediate response to SMT in their practice. This was despite them answering positively to the question in the formative information stage. Consequently 16 responses were analysed. Fifteen possible predictor items were excluded after Round 1 because they did not achieve the threshold value of at least 75% agreement (Table 2). Seven additional possible signs or symptoms were suggested from Round 1 responses and were added into the survey for Round 2 (Table 2). These items included aspects of a therapeutic alliance namely, the patient having trust and confidence in the practitioner, the practitioner understanding the patient expectations and goals and the presence of a comprehensive history. Also included was the presence of a cavitation during treatment, reproduction of symptoms at the end of ranges of motion or on spinal springing and the patient being susceptible to placebo.

The mean estimated score of the percentage of patients who participants believed experienced instantaneous relief from SMT approached 40% (SD 27.5%) and ranged from a low of 10% to a high of 80%. Round 2 responses produced highly similar results for this question and consequently was not included in Round 3.

#### Round 2

In Round 2, 17 participants responded, and one practitioner was excluded as they also had not observed an immediate beneficial response to SMT in practice. The responses were analysed and a further 28 variables were excluded (Table 2) on the same basis that they did not achieve at least 75% agreement of being “slightly” or “most predictive”. Consequently 19 variables were included for Round 3.

#### Round 3

In Round 3, there were 17 participants, none were excluded. The highest predictor of instantaneous relief from NSLBP following SMT was “a history including a good response to previous spinal manipulation” (Table 2). This was the only predictor to attain a score of 75% agreement of being “most predictive”. There was only one item that did not meet the 75% agreement criterion of being “slightly” or “most predictive”, which was “taking a comprehensive history” and was therefore removed resulting in a final list of 18 items. The predictive scores for each item was then calculated (mean score, SD, sum and ranges) and are seen in Table 3. Many of the items appeared to cluster together and represented distinct domains that were clinically relevant and interpretable. After a post hoc discussion among the authors, the items were placed into five domains: Patient factors; Practitioner factors; Patient signs and symptoms; Instrument of measurement; Presence of cavitation with SMT.

**Table 3:** Final 18 predictor items placed in 5 Domains ranked by mean score and sum

Predictors	Mean	Std. Deviation	Sum	Range	Minimum	Maximum
**Patient factors**						
Patient history of a good response to previous SMT	1.88	0.33	32	1	1	2
Patient has trust and high confidence in the practitioner	1.35	0.49	23	1	1	2
Patient susceptible to placebo effect	0.94	1.03	16	4	-2	2
Patient has a comprehensive understanding of condition	0.76	0.90	13	3	−1	2
**Practitioner factors**						
Good patient-practitioner relationship	1.35	0.49	23	1	1	2
Professional opinion of health status - excellent/ very good	1.18	0.73	20	3	−1	2
Practitioner understanding of patient expectations and goals	1.06	0.66	18	3	−1	2
Professional opinion of health status – good	0.88	0.99	15	3	−1	2
**Signs and symptoms of NSLBP presentation**						
Duration of symptoms < 16 Days	1.06	1.09	18	4	−2	2
Pain improves with exercise, but not rest	1.06	1.09	18	4	−2	2
No symptoms in the lower extremities	1.00	0.87	17	4	−2	2
Patient has an acute condition (< 14 days)	0.94	1.20	16	4	− 2	2
No symptoms distal to the knee	0.94	1.20	16	4	−2	2
Decreased active range of motion	0.76	1.09	13	4	−2	2
Decreased passive range of motion	0.76	1.09	13	4	−2	2
Close reproduction of symptoms on spinal springing and/or end range loading	0.65	1.32	11	4	−2	2
**An instrument of measurement (FABQ)**						
Fear Avoidance Beliefs Questionnaire work scale score less than 19 out of a high of 42	0.82	0.95	14	4	−2	2
**The presence of a cavitation following SMT**						
The production of a the clicking sound (cavitation) at the moment of thrust	0.71	1.21	12	4	−2	2

### Interpretation of non-consensus

The items that were not included in Round 1 and 2 (did not reach consensus), were indicative of a patient with a significant, chronic and disabling LBP. The items included persistent and highly rated pain, the presence of psychosocial factors (depression / anxiety / stress), and pain on coughing sitting and at night. Items that did not fit this profile were gender, and any specific age range.

The non-consensus items not included in Round 3 were again indicative of a more chronic LBP patient presentation. Items such as subacute, pain with motion (sit, stand, run, walk, flex, extend), obesity, and morning gel phenomena were rated poorly as likely predictors of instant response. However, some non-consensus items appeared not to match the profile of a chronic LBP patient and included low ratings of pain severity, a poor response to NSAIDs, and lumbar spine hypomobility.

## Discussion

This is, to our knowledge, the first study to seek the opinion of experts’ in HVLA SMT as to factors that may predict a person who will have an immediate positive response. We presented a total of 62 items to 17 experts, which was ultimately reduced to 18 items using a modified Delphi methodology. These items were able to be allocated into 5 clinically relevant domains.

It appears logical that the most highly rated predictive factor for a patient to experience an instantaneous positive response to HVLA SMT for NSLBP was “a history that included a good response to previous spinal manipulation”. In support of this opinion is previous research that showed an immediate response on the first consultation was highly predictive of the outcome at the fourth consultation [22]. Also, a good response to prior SMT identified patients most likely to respond to maintenance care for NSLBP [30]. Finally, if a patient has had a previous positive experience then their expectations would more likely be for a similar result and such expectations are known to be predictive of treatment outcomes [31, 32].

The items, “A patient with high trust and confidence in the practitioner”, “a good patient-practitioner relationship”, “patient has comprehensive understanding of a condition” and “practitioner understanding of patient expectations” were all highly agreed upon predictors and reflect known aspects of a therapeutic alliance [33]. A high-quality therapeutic alliance has been shown to be a significant contributor to the outcomes of patients with NSLBP undergoing manual therapy [34–36]. Some have suggested that the therapeutic alliance plays a mediating role in patient outcomes [34]. If so, then this Delphi survey constructed item list may be identifying aspects of the practitioner-patient interaction that maximizes the impact of the therapeutic alliance when a manual therapist is using HVLA SMT as an intervention, and as such warrant’s further investigation.

There were two items in the final list that were in accord with factors known to impact negatively on outcomes of patients experiencing LBP. These were the presence of co-morbidities [37, 38] (“opinion of health status”) and psychosocial factors [39] (“FABQ Work scale”). If the practitioner identifies that there are no “Yellow flags” then time consuming and complex interventions can be avoided, thus “streamlining” the clinical encounter to increase efficiencies of care. This also increases the likelihood of avoiding unnecessary labels surrounding “psychosocial” complexities that many manual therapists feel inadequately prepared to deal with [40].

The items of “patient susceptible to placebo effect” and “the production of a clicking sound (cavitation) at the moment of thrust”, although not linked by the experts in this study, could possibly be viewed together. The thrust component without a cavitation can reduce spinal pain [41]. However, there is also evidence that, at least in part, the audible sound produces a placebo effect [42]. This is a psychobiological phenomenon where many mechanisms are at play [43, 44]. A patient’s response to such placebos are well documented and a range of contextual factors have been identified that maximize its effect [45]. This study suggests practitioners who use HVLA SMT believe this has a role to play in patients who obtain immediate relief. Other items on this final list, such as patient expectations previously discussed, also contribute to contextual factors [44]. This raises the question of “Do patients who experience this type of response to HVLA SMT also experience it in other clinical settings or for other health care interventions?” Studies have suggested that the personality traits of optimism, in tandem with low state anxiety, are predictive of a placebo effect [46]. However, research regarding the correlation between personality traits and placebo effects is not consistent, and an individual’s expectations appear to play a larger role [47]. A simpler starting point to answer this question, and the role of non-mechanical factors in general in the NSLBP subgroup, may be to conduct qualitative studies seeking the views of patients known to have this type of dramatic response.

While many items were thought to be impacted on by psychosocial factors there was nonetheless a considerable number with a biomechanical focus. Three of the five criteria for the clinical prediction rule for identifying patients with non-radicular low back pain who will benefit from SMT [48] were retained and indicated a milder less complicated profile i.e., pain shorter in duration, no radiculopathy or fear-avoidance issues, and reduced or painful (end) ranges of motion. While there is some evidence that patient self-reported changes in motion were predictive of post-SMT immediate improvement [49], the reliability and validity of altered ranges of movement or pain provocation is yet to be demonstrated in clinical trials and is thought to require advanced studies to inform their clinical utility [50–52]. Also, currently the diagnosis of LBP has moved toward clusters of tests [53]. Taken in combination, the findings from this study suggest that experts in HVLA SMT are of the view that this novel cluster of biomechanical factors are worthy of further testing for reliability and validity.

The expert HVLA SMT practitioners demonstrated a wide range of estimates of likely numbers of patients who had an immediate strong response to SMT. Interestingly three practitioners reported never having seen it in their clinical experience. Recent thinking for LBP has reconceptualised it as a recurrent persistent condition, somewhat like asthma, that can better be described in terms of variable trajectories [54]. While it is interesting to speculate on the trajectory and the numbers of patients who are perceived to have an immediately strong response to SMT, ultimately this will be decided by longer-term follow-up studies.

### Strengths and limitations of the study

A recognised Delphi methodology [26] was used to obtain the opinion of experts in the field on a single outcome anecdotally recorded in practice. All experts had a sufficient level of experience (at least 5 years of clinical experience) [25] and were derived from both the chiropractic and manipulative physiotherapy professions who regularly employ HVLA SMT as an intervention. Additionally, the snowball sampling resulted in a high number of Australian participants. Nonetheless, this study involved 19 participants and this sample size raises questions of generalizability.

Also, there is considerable debate about the ability of subgroup analyses to examine treatment-effect modification across NSLBP subgroups defined by patient characteristics [55]. Factors thought to contribute to this uncertainty are poor methodological quality, the absence of a clearly established biological rationale, and heterogeneity of treatment effects [55]. These factors should be borne in mind if attempts are made to further explore the findings of this preliminary investigative study.

Another limitation is the assumption that an immediate response post-SMT is a phenomenon that has objective properties, such as signs and symptoms, that can be used for identification / quantification purposes. The observations of such a response have been derived anecdotally, and the definition of “instantaneous relief” will likely differ among practitioners.

Despite these obvious limitations there is significant evidence that a three-round Delphi such as the one conducted can be successful in establishing its purpose [26].

A successful Delphi effectively identifies differing opinions, acts as an efficient group communication tool to deal with topics of complexity and/or uncertainty, and has the ability of establishing a homogeneous expert opinion or result [25].

### Future research

The 18 factors identified in this study could be formed into an instrument of measurement and then tested for reliability and validity. Such a future study would need to include a variety of patients of differing ages, gender and severity of problem/ condition.

Several of the factors are novel and previously unexplored when seeking to predict patient outcomes to SMT, in particular those related to trust and confidence in the practitioner and susceptibility to placebo. As such they may warrant preliminary investigations before progressing with further subgrouping studies using these 18 items.

## Conclusion

Developing a well-informed decision-making tool regarding the most appropriate manual therapy and treatment strategy for an individual is an admirable goal. Success in this regard would potentially save time, reduce costs, improve treatment outcomes and reduce adverse events from unnecessary treatment.

The 18 factors identified in this study can theoretically be used to create an instrument of measurement that may be used clinically to predict those NSLBP patients who will receive instantaneous relief post-SMT. Future research on these factors and their reliability and validity is recommended.

## Supplementary information

**Additional file 1.** Round 1 Initial Survey

## Data Availability

Not applicable.

## Abbreviations

HVLA: High-velocity low-amplitude
LBP: Low back pain
NSLBP: Non-specific low back pain
SMT: Spinal manipulation therapy
