# Supplementary figures and images for: Predictors of instanteous relief from spinal manipulation for non-specific low back pain: a delphi study
Source: Chiropr Man Therap. 2020 Jul 2;28:39. doi: 10.1186/s12998-020-00324-7 (PMC7331174; doi:10.1186/s12998-020-00324-7)

# Appendix 1: Round 1 Initial Survey


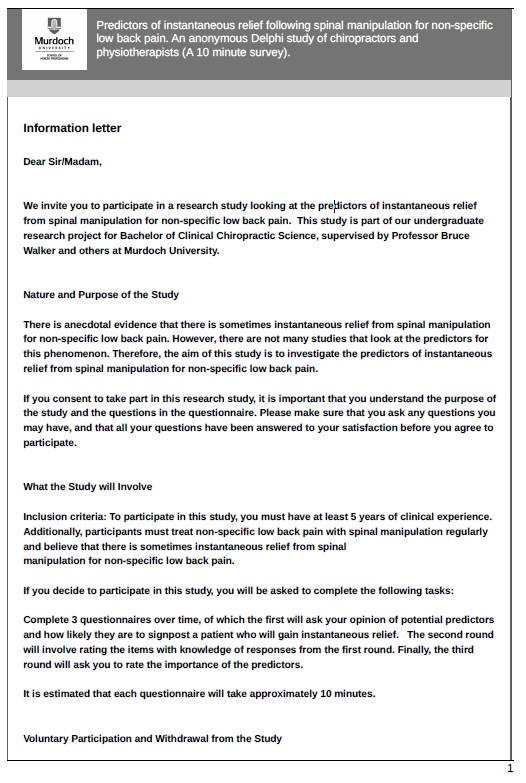


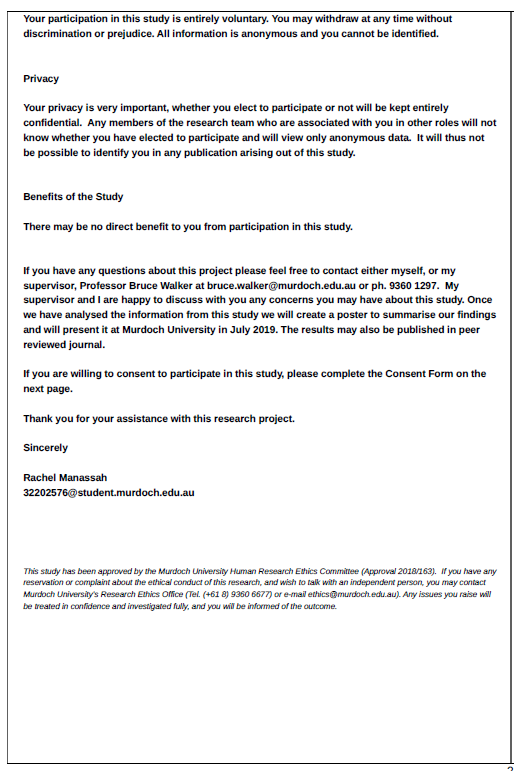


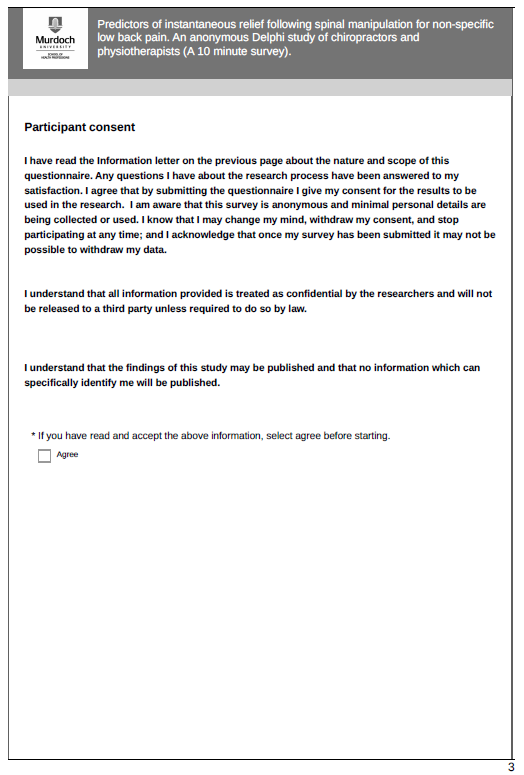


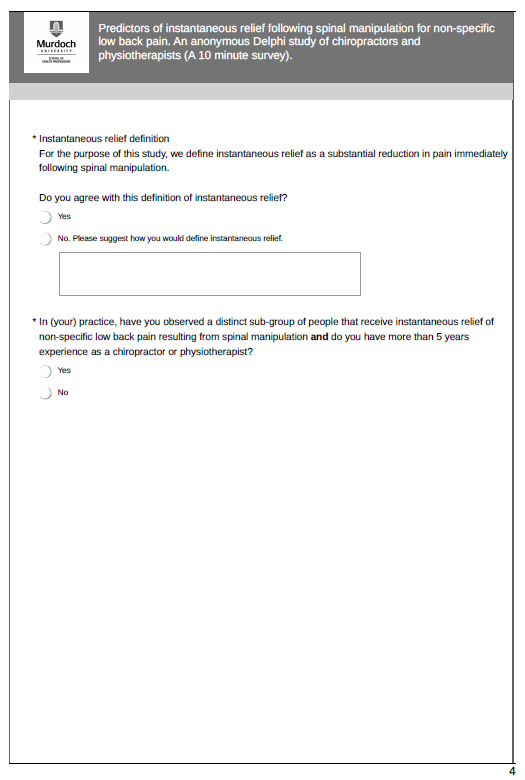


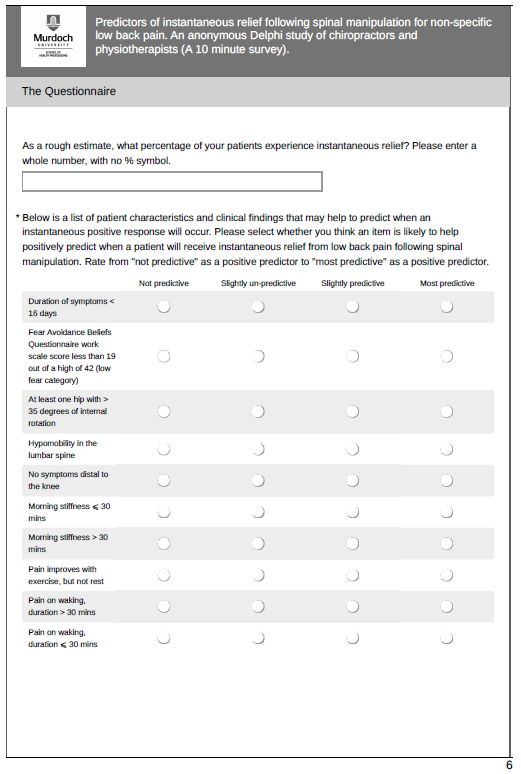


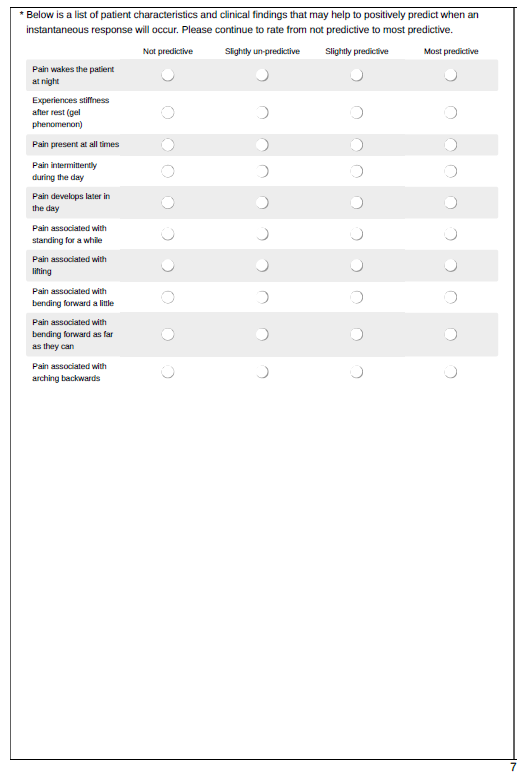


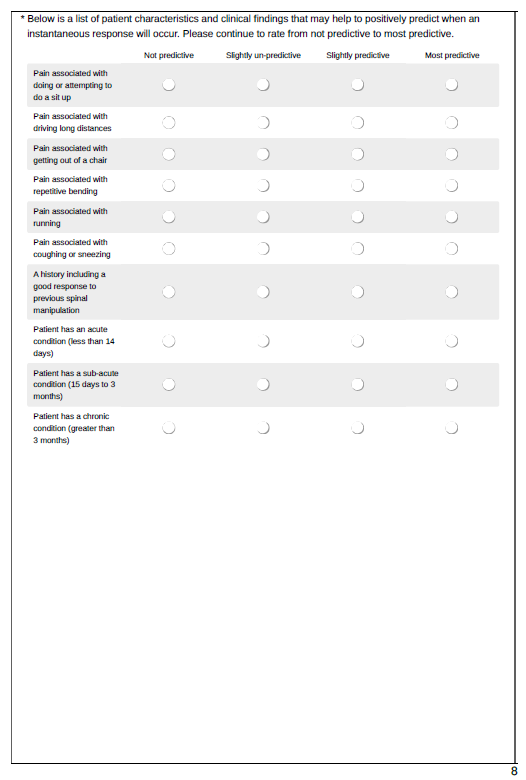


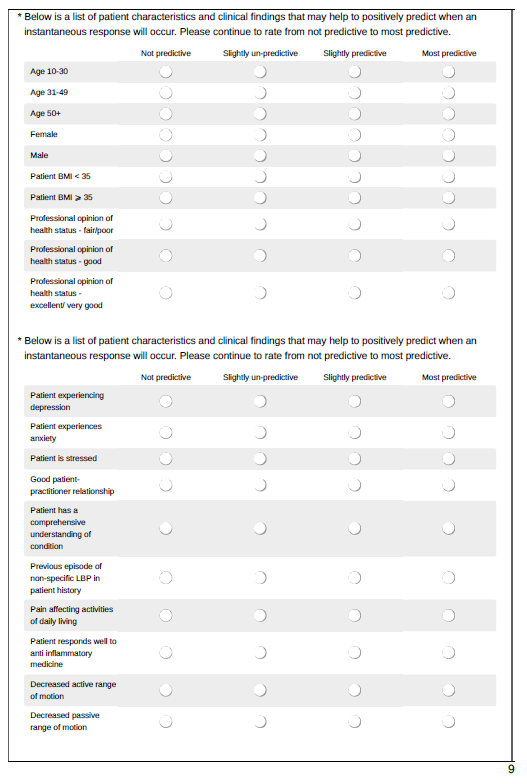


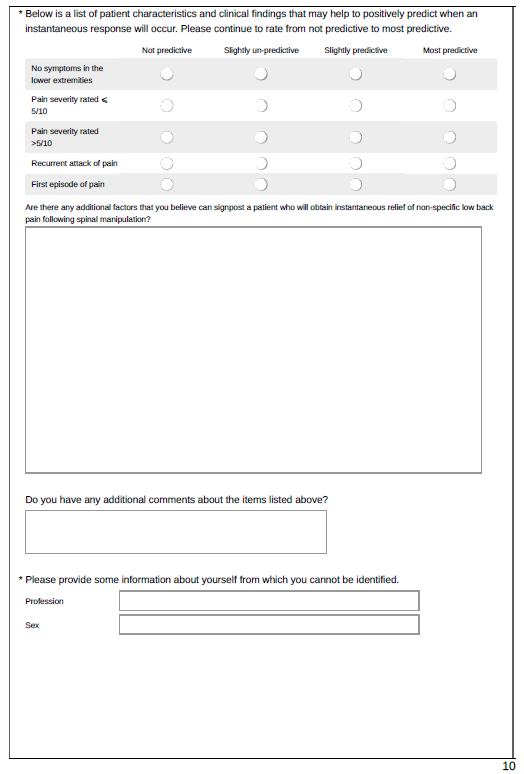

Supplement: Supplementary file 1 — Additional file 1. Round 1 Initial Survey [file 12998_2020_324_MOESM1_ESM.docx]
